# Childhood adversity and recurrence of psychotic experiences during adolescence: the role of mediation in an analysis of a population-based longitudinal cohort study

**DOI:** 10.1017/S003329172200071X

**Published:** 2023-07

**Authors:** N. Dhondt, L. Staines, C. Healy, M. Cannon

**Affiliations:** 1 Department of Psychiatry, Royal College of Surgeons in Ireland, Dublin 2, Dublin, Ireland; 2 St James's Hospital, Dublin 8, Dublin, Ireland; 3 Department of Psychiatry, Beaumont Hospital, Dublin 9, Dublin, Ireland

**Keywords:** Psychotic experiences, psychotic-like experiences, childhood adversity, mediation analysis, self-concept

## Abstract

**Background:**

Psychotic experiences (PEs) are relatively common in childhood and adolescence and are associated with increased risk of functional issues and psychiatric illness in young adulthood, and PEs that recur are associated with increased risk of poorer psychiatric and functional outcomes. Childhood adversity is a well-established risk factor for PEs. The aim of this study was to investigate (1) the relationship between childhood adversity and recurring PEs in adolescence and (2) candidate mediators of that relationship.

**Methods:**

We used data from Cohort ‘98 of the Growing Up in Ireland study (*n* = 6039) at three time points (ages 9, 13 and 17) to investigate the relationship between childhood adversity (parent-reported at age 9), recurring PEs (measured using a subset of the Adolescent Psychotic-like Symptoms Screener at ages 13 and 17). The mediating roles of parent–child relationship, internalising and externalising difficulties, self-concept, physical activity, dietary quality, perceived neighbourhood safety and friendship quantity were investigated using the KHB path decomposition method.

**Results:**

Childhood adversity was associated with an increased risk of recurring PEs with a population attributable fraction of 23%. Internalising difficulties and self-concept explained 13% of the relationship between childhood adversity and PEs suggesting a partial mediation. A significant direct effect remained between childhood adversity and recurring PEs.

**Conclusions:**

The established relationship between childhood adversity and PEs may be mainly driven by the relationship between childhood adversity and recurring PEs. Internalising difficulties and self-concept together mediate part of the relationship between childhood adversity and recurring PEs.

## Introduction

Psychotic experiences (PEs) have an estimated lifetime prevalence of around 4–9% and are more common in childhood and adolescence (Kelleher et al., 2012; Maijer, Begemann, Palmen, Leucht, & Sommer, 2017). PEs in childhood and adolescence are associated with a range of negative outcomes including poorer global functioning in adulthood and an increased risk of psychiatric disorders (Healy et al., 2018, 2019a). The majority of PEs are transient, but they may recur (often described as being ‘persistent’) in 20–40% of cases (Kalman, Bresnahan, Schulze, & Susser, 2019; van Os, Linscott, Myin-Germeys, Delespaul, & Krabbendam, 2009). Adolescents with transient PEs have been found to have an increase in interpersonal and educational or vocational difficulties during adolescence that do not persist into young adulthood, and there is mixed evidence of their association with later psychopathology (Coughlan et al., 2021; Downs, Cullen, Barragan, & Laurens, 2012). Conversely, PEs that recur are associated with increased psychopathology on follow-up and increased risk of subsequent non-suicidal self-injury and suicide attempts (Hielscher et al., 2021; Yates et al., 2010). Recurrence of PEs is also associated with increased risk of subsequent clinical psychosis (Dominguez, Wichers, Lieb, Wittchen, & van Os, 2011).

Childhood adversity is a broad term for traumatic and stressful experiences in early life, covering a range of experiences of varying prevalence, from abuse, neglect and exposure to natural disasters to bullying, with as many as half the population reporting at least one childhood adversity and nearly a third reporting three or more (Green et al., 2010; Merrick, Ford, Ports, & Guinn, 2018). Childhood adversity in both prospective and retrospective reports has been found to predict adult psychopathology (Green et al., 2010; Newbury et al., 2018). The evidence on differing baseline characteristics between those with transient and recurring PEs is inconclusive, but childhood adversity is one of the few well-established risk factors for PEs and has been associated with increased risk of PE recurrence (Croft et al., 2018; Kalman et al., 2019; Trotta, Murray, & Fisher, 2015; van der Steen et al., 2019; Varese et al., 2012). Social support and physical activity have been found to be protective against PEs among those with exposure to adverse experiences in adolescence (Crush et al., 2018). Positive attributes (including positive personality traits and adaptive social behaviours) may reduce the risk of recurring PEs in those who have experienced childhood adversity (Pan et al., 2019).

Mediation analysis is the investigation of third variables that account for at least part of the causal pathway between an exposure and an outcome, providing a mechanism (a how or why) by which the exposure may lead to a given outcome (Baron & Kenny, 1986). There may be a direct pathway between the exposure and the outcome, and either additionally or instead, depending on the strength of the mediator, an indirect path via the mediator's impact (Baron & Kenny, 1986). In this way, establishing mediators between childhood adversity and PEs may explain the mechanism by which adversity increases risk. A review of mediators between childhood adversity and psychotic symptoms identified a number of mediators that they categorised into five ‘families’: post-traumatic sequelae, affective disturbance and dysregulation, cognitive processes, appraisal of subsequent stressors and life circumstances and exposure to other psychosis risk factors (Williams, Bucci, Berry, & Varese, 2018). In addition to psychological mediators, engagement in physical activity, greater neighbourhood social cohesion and social support have been found to be protective against PEs in adolescence among those exposed to poly-victimisation (Crush et al., 2018). Different kinds of adversity may be mediated in different ways, as depressive symptoms have been found to mediate between exposure to domestic violence and PEs, while external locus of control was found to be a more important mediator between bullying and PEs (Fisher et al., 2013). To date, there is little knowledge about specific mediators for recurring PEs.

The present study investigates the relationship between childhood adversity and transient and recurring PEs, and uses mediation analysis to compare the contributions of a number of different factors to this pathway. Using data from a large nationally representative cohort we examined whether childhood adversity predicted recurring PEs at age 13 and 17, and whether any of a number of socio-emotional factors mediated the relationship between childhood adversity and recurring PEs.

## Methods

### Participants

Cohort ‘98 (also known as the ‘child cohort’) of the Growing Up in Ireland study comprises a representative sample, initially consisting of 8658 children and their families, in 910 primary schools (Thornton, Williams, McCrory, Murray, & Quail, 2016). 50% of those invited to participate in 2007 and 2008 were recruited, and 75% of those who participated at age 9 also participated in the follow-up at age 17 (81% of those who had participated at age 13). The sample who participated at all three time points consisted of 6039 children and their families (Murphy, Williams, Murray, & Smyth, 2019). Questionnaires and interviews were undertaking with participating children and their families at each wave. To account for attrition between the waves, the data has been reweighted with respect to differential response characteristics for each time point (Murphy et al., 2019).

### Ethical considerations

The GUI received ethical approval from the Health Research Board's research ethics committee in Ireland. Ethical Approval for this secondary analysis was granted by the Research Ethics Committee of the Royal College of Surgeons in Ireland (REC1486).

### Exposure

In this study, we continued to use the construction of childhood adversity used in our previous analysis of childhood adversity and adolescent psychopathology, changed to include the deaths of siblings as a severe life event due to new access to this data (Dhondt, Healy, Clarke, & Cannon, 2019). This measure is based on parent-report questions about 14 stressful life events. The child was considered to have experienced adversity if their primary caregiver reported one of seven ‘severe’ events: the death of an immediate family member, the death of a close friend, having a parent in prison, drug taking or alcoholism in the immediate family, mental disorder in the immediate family, serious illness or injury, a stay in a foster home or residential care, or if they reported three or more of the remaining seven events: divorce or separation of their parents, the death of a close family member, serious illness or injury of a family member, conflict between parents, moving to a new house, moving to a new country or another unspecified event.

In a previous paper, we have compared this childhood adversity measure to alternative measures using such as cumulative CA and a latent measure derived from item-response theory and found little difference between the measures (Healy et al., 2021). We have repeated this analysis (see Online Supplementary Materials) and continue to find similar results with all three measures.

### Mediators

We based our selection of mediators on the families of mediators identified in the review by Williams et al. (2018), namely post-traumatic sequelae, affective disturbance and dysregulation, cognitive processes, appraisal of subsequent stressors and life circumstances and exposure to other psychosis risk factors (Williams et al., 2018). We attempted to find mediators in the age 9 data that would provide information on each of these categories, but two were unavailable, namely: post-traumatic sequelae (with no age 9 measure of post-traumatic symptoms or dissociation) and exposure to other psychosis risk factors (substance misuse was not assessed at age 9).

### Affective disturbance and dysregulation

These were measured from the parent-report Strengths and Difficulties Questionnaire (SDQ), using the externalising and internalising subscale cut-offs (Goodman, 2001). The SDQ is parent-reported, but no child-reported alternative was available at age 9.

### Cognitive processes

Cognitive processes assessed included self-concept and positive coping mechanisms including social behaviours, physical health, and dietary health.

Self-concept is a construct referring to the many facets of an individual's beliefs and knowledge about their own personal attributes and qualities, and has been found to be strongly related to PEs (Healy et al., 2019b). In this study self-concept was measured using the Piers-Harris scale, a 60-item self-report questionnaire designed for ages 7–18, based on a ‘yes’ or ‘no’ answer to statements regarding their self-perception (Piers & Herzberg, 2002). While the scale includes six subscales on behavioural adjustment, intelligence and school status, physical appearance and attributes, freedom from anxiety, popularity and happiness and satisfaction, for simplicity and because prior research on the fine-grained relationship between self-concept and PEs has already been undertaken, we only used the ‘total’ score in this study (Healy et al., 2019b).

Social behaviours were measured using a parent-reported question on how many friends the child had, with the cut-off at 2 or more (the 92^nd^ percentile).

Childhood adversity has been associated with subsequent health risk factors including obesity and physical inactivity (Felitti et al., 1998). We included measured physical health risk factors investigated as mediators under the umbrella of ‘cognitive processes’ in line with research on health behaviours that has found, along with biological and demographic characteristics, individual psychological characteristics including beliefs, motivations and cognitions affect these choices (Baumann et al., 2012). While children have less choice about these behaviours than adults, children's preferences have been found to influence dietary intake, and are likely to play a role in the extent of physical exercise participation (Mazarello, Ong, & Lakshman, 2015).

Increased participation in physical exercise has been found to be protective against PEs in those who have experienced victimisation (Crush et al., 2018). Physical exercise was assessed with a question on the frequency of engagement in hard exercise for twenty minutes or more in the last 2 weeks. The threshold was 9 of the previous 14 days, as a proxy for WHO guidelines (*WHO guidelines on physical activity and sedentary behaviour*, 2020).

Diet was included based on evidence identifying increased fruit and vegetable consumption as a protective factor against psychosis, and refined carbohydrates as a risk factor (Aucoin, LaChance, Cooley, & Kidd, 2020). While dietary choices are not solely determined by children's selections, in most families children's preferences may contribute to diet, and though at age 9 are not likely to be making dietary choices with nutrition at the forefront of their minds, we considered diet as a possible cognitive process mediator due to evidence that worse dietary choices are made in the context of increased cognitive load, and because the choice of unhealthier comforting foods may be a stress coping strategy (Michels et al., 2012). Diet was assessed in the GUI with parent- and child-based questions on intake of specific food items in the prior 24 h. Sum scores of three measures of fruit and vegetable, and refined carbohydrate consumption were created and then z-score standardised. We selected this sum score based on previous work in the GUI using a dietary quality score, with the focus modified onto only the items that may be related to PEs (Perry et al., 2015).

### Subsequent stressors and life circumstances

These were assessed using the parent-reported conflict and positivity scales from the Pianta parent-child relationship scale (Pianta, 1992). A question on perceived safety in the child's home neighbourhood (‘Do you feel safe living around here’) was included and could also be considered as part of the appraisal of subsequent stressors and life circumstances.

### Outcome

PEs in the GUI are assessed at ages 13 and 17 by six of the seven questions taken from the Adolescent Psychotic-Like Symptom Screener (Kelleher, Harley, Murtagh, & Cannon, 2011). This was scored based on half a point for a ‘maybe’ and a full point for a ‘yes’, with a score over two being taken as positive for PEs, or a definite ‘yes’ response to the question on auditory hallucinations, which has been found to be a valid single-item measure of PEs. The APSS consists of the following six questions: have you ever heard voices or sounds that no-one else can hear; have you ever seen things that other people could not see; have you ever thought that people are following you or spying on you; some people believe that their thoughts can be read by another person. Have other people ever read your mind; have you ever felt that you were under the control of some special power; have you ever felt that you have extra-special powers?

One item from the APSS is not used in the GUI (‘Have you ever had messages sent to you through TV or Radio’) and has previously been found to have only 40% positive predictive value for any type of psychotic experience (Kelleher et al., 2011). The APSS has been validated against clinical interviews and has been shown to have good sensitivity and specificity for PEs.

Recurrent was defined as meeting the criteria for PEs at both time-points (ages 13 and 17) and was included to look at the differences between those who had transient PEs (at age 13 only) and those who had recurring experiences at both timepoints.

### Confounding variables

A number of variables were included as confounders as they were considered to have an effect on both childhood adversity and PEs, including age, gender, socio-economic status (measured as income quintile and maternal education level), minority status and urbanicity. These were controlled for in all analyses.

### Statistical analyses

Mediation analysis was chosen due to the potential for clarifying mechanisms of development and therefore identifying means of reducing the risk on the pathway to PEs and subsequent psychopathology in those who have already experienced childhood adversity. We undertook the standard steps of mediation analysis to (1) establish whether there was an overall relationship (i.e. the total effect) between the exposure (childhood adversity) and the outcome (PEs) (2) establish whether the exposure predicted our candidate mediators and (3) whether the candidate mediators predicted the outcome (Baron & Kenny, 1986).

Logistic regressions were used to investigate whether childhood adversity at age 9 and possible confounds predicted PEs at ages 13 and 17. Linear and logistic regressions were used to investigate the relationship between childhood adversity and the proposed mediators at age 9, and between the mediators at age 9 and PEs at ages 13 and 17 (see Table 1).

**Table 1.** Age 9 mediators and their associations with childhood adversity and recurring PEs

	Prediction by childhood adversity OR (95% CI)	Prediction of reoccurring PEs OR (95% CI)	Univariate indirect effect OR (95% CI)	% accounted for by mediators	Multivariate indirect effect OR (95% CI)	% accounted for by mediators
Candidate mediators associated with both exposure and outcome						
Internalising	**1.68** (**1.29–2.20)**	**3.25** (**1.87–5.64)**	**1.05 (1.01–1.10)**	**6.87%**	**1.05 (1.03–1.07)**	**7.01%**
Self-concept	**0.17** (0.08–0.26)*	**1.34** (**1.11–1.62)**	**1.06 (1.011.11)**	**7.45%**	**1.04 (1.02–1.07)**	**5.96%**
Externalising	**1.64** (**1.22–2.08)**	**2.61** (**1.30–5.28)**	1.04 (1.00–1.09)	5.45%	–	–
Parent-child conflict	**0.26** (0.17–0.34)*	**1.24** (**1.03–1.50)**	1.04 (0.99–1.09)	5.14%	–	–
Having more than one friend	**0.56** (**0.42–0.75)**	0.53 (0.27–1.01)	–	–	–	–
Candidate mediators not associated with both exposure and outcome						
Parent-child positivity	0.03 (−0.05 to 0.11)*	1.02 (0.82–1.28)	–	–	–	–
Exercise frequency	1.05 (0.88–1.25)	1.15 (0.74–1.79)	–	–	–	–
Fruit and vegetable consumption	−0.04 (−0.12 to 0.04)*	0.84 (0.68–1.02)	–	–	–	–
Refined carbohydrate consumption	−0.01 (−0.09 to 0.04)*	1.21 (0.99–1.49)	–	–	–	–
Neighbourhood safety	0.64 (0.42–0.99)	**0.21** (**0.11–0.41)**	–	–	–	–

OR, Odds ratio; CI, Confidence interval.

*Asterisks indicate linear regression coefficients as opposed to ORs.

Bolded statistics are significant at the *p* < 0.05 level, with the Benjamini-Hochberg false discovery rate correction applied.

As in our previous research, we used the Karlson, Holm and Breen method (the *khb* package in Stata) to compare estimated coefficients of nested non-linear probability models in Stata (version SE 15.0) for Windows (Dhondt et al., 2019; Kohler, Karlson, & Holm, 2011). The *khb* package allows the decomposition of a total effect into an indirect effect (that part of the relationship explained by mediators) and a direct effect (the relationship remaining once mediators are accounted for) and provides coefficients that are comparable even in cases where mediating variables have independent effects on the dependent variable, as is possible in this case. We used logistic regressions with *khb*.

Due to the weighted nature of our sample it was not possible to use bootstrapping with this analysis – individuals could not be resampled due to their variable contributions to the analysis.

## Results

### Prevalence and demographics

Once reweighting was applied, 1748 (29%) participants reported childhood adversity at age 9. Regarding the prevalence of PEs, 738 (12.8%) participants reported PEs at age 13, and 580 (9.8%) at age 17. 4645 (80.9%) participants did not report PEs at either age 13 or age 17 and 175 participants (3.8%) reported recurring PEs, i.e. at both ages 13 and 17.

Table 2 shows the demographic characteristics of the participants who had or had not experienced childhood adversity, and who reported recurring PEs as compared with those who never reported any PEs. Not having Irish citizenship, living in an urban area and having lower socio-economic status, in terms both of income quintiles and primary caregiver's highest level of education were associated with an increased risk of childhood adversity. Those with recurring PEs were less likely to have Irish citizenship.

**Table 2.** Demographic characteristics of those with and without childhood adversity and recurring psychotic experiences (PEs)

	Overall (*n* = 6039)	Non-exposed to childhood adversity (*n* = 4291)	Exposed to childhood adversity (*n* = 1748)	OR (95% CI)	Controls (*n* = 4479)	Recurring PEs (*n* = 175)	OR (95% CI)
Gender (% female)	49.13	47.89	52.17	0.84 (0.75–0.94)	49.32	52.96	0.86 (0.58–1.30)
Age in years (% age 9 at first wave)	98.27	98.36	98.05	0.87 (0.57–1.33)	98.26	98.11	1.76 (0.61–5.05)
Income quintile							
Lowest	18.17	**16**.**88**	**21**.**29**	**1.28** (**1.06–1.53)**	17.86	38.10	0.74 (0.41–1.37)
Second	20.98	**19**.**3**	**25**.**06**	**1.31** (**1.10–1.56)**	20.16		
Third	20.44	20.5	20.28	Reference	20.21	27.23	Reference
Fourth	20.26	**21**.**43**	**17**.**41**	**0.82** (**0.68–0.99)**	21.34	34.68	0.62 (0.35–1.07)
Highest	20.15	**21**.**89**	**15**.**95**	**0.74** (**0.61–0.89)**	**20**.**43**		
Primary caregiver's highest level of education							
None/primary only	5.62	**4**.**51**	**8**.**35**	**2.25** (**1.78–2.85)**	5.00	*	NS
Junior cert or equivalent	23.54	**22**.**23**	**26**.**73**	**1.46** (**1.26–1.69)**	**23**.**16**	*	**Significantly fewer with PEs**
Leaving cert or equivalent	37.07	39.09	32.13	Reference	37.31	45.27	Reference
Postsecondary diploma/cert	16.46	**16**.**43**	**16**.**52**	**1.22** (**1.03–1.45)**	17.14	*	NS
Primary degree	11.18	11.37	10.72	1.15 (0.95–1.39)	11.15	*	NS
Postgraduate degree	6.13	6.37	5.55	1.06 (0.83–1.36)	6.24	*	NS
Irish citizenship	95.52	**96**.**51**	**93**.**09**	**0.49** (**0.38–0.62)**	**95**.**91**	*	**Significantly fewer with PEs**
Region (% urban)	43.98	**43**.**12**	**46**.**08**	**1.13** (**1.01–1.26)**	43.08	50.72	1.36 (0.90–2.05)

OR, Odds ratio; CI, Confidence interval; NS, Non-significant.

Bolded statistics indicate significant differences between groups.

*Asterisks indicate cells that would indicate fewer than 30 individuals, which cannot be disclosed from the restricted microdata file of the GUI for the risk of identifying participants.

### Childhood adversity and recurring PEs

Of those who reported recurring PEs, 78 (45%) reported childhood adversity at age 9, compared with 267 (30%) of those with transient experiences at either age 13 or age 17 only, and 1225 (27%) of those with no PEs. A multinomial logistic regression between childhood adversity at age 9 and those who had transient, recurring or no PE, controlling for confounders, found that when those without PEs were taken as the reference group, transient PEs were not associated with childhood adversity (OR 1.17, 95% CI 0.99–1.38) and recurring PEs were associated with childhood adversity (OR 2.13, 95% CI 1.54–2.95). When comparing those with recurring PEs to those without PEs, childhood adversity had a population attributable fraction of 23%. Taking transient PEs as the reference category, childhood adversity was not significantly associated with having no PEs (OR 0.86, 95% CI 0.73–1.01) and childhood adversity was significantly associated with an increased risk of having recurring PEs (OR 1.83, 95% CI 1.29–2.60).

### Mediation analysis

**Fig. 1.**
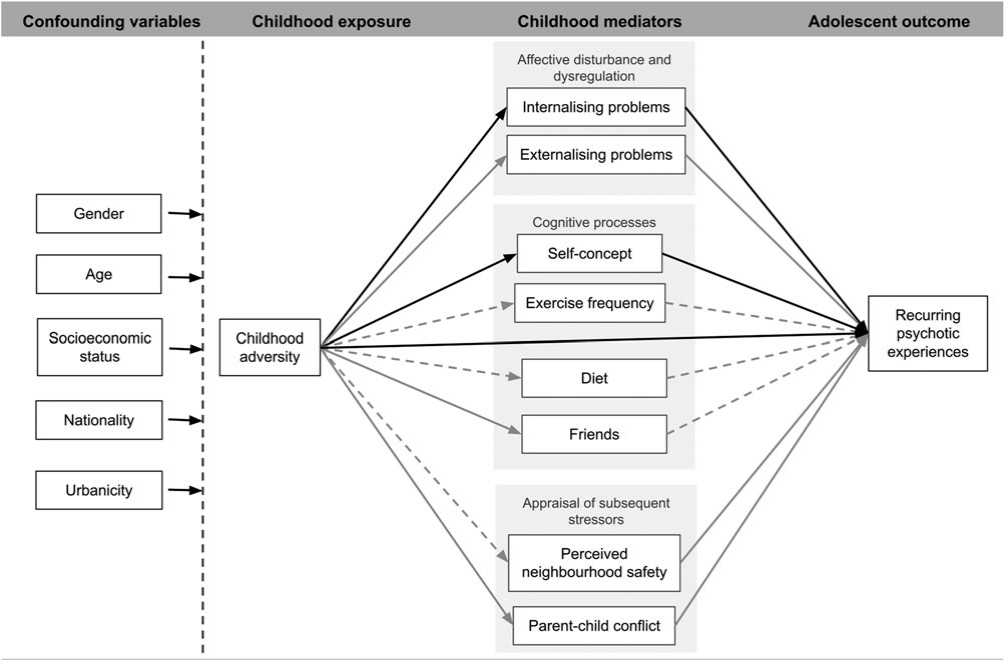
Proposed relationship between confounders, childhood exposure and mediators and adolescent outcomes. Dotted lines show relationships proposed but not found, lighter coloured lines indicate variables related to exposure and outcome but not explaining a significant portion of the indirect effect. Black lines indicate significant direct and partially mediating relationships.

Figure 1 shows the proposed relationship between confounders, candidate mediators, childhood adversity and outcomes. Table 1 shows the relationship found between exposure, mediators and outcome. Childhood adversity was associated with: increased internalising and externalising difficulties; lower scores on the self-concept scale; having one or no friends; increased conflict with parents and feeling less safe in their home neighbourhood. It was not associated with the positive parent-child relationship, exercise participation or dietary intake (higher fruit and vegetable consumption or lower refined carbohydrate consumption). Compared to not having PEs at all, recurring PEs were associated with: internalising and externalising difficulties; lower self-concept scores; more conflict with parents and feeling less safe in their home neighbourhood in childhood.

A correlation analysis was undertaken to assess collinearity between the mediators. All of the mediators were significantly related to one another except: carbohydrate consumption with feeling safe in the neighbourhood and having friends; feeling safe in the neighbourhood and internalising difficulties and exercise participation, and externalising difficulties and exercise participation. (see Online supplementary Table S7). However, aside from parent-child conflict and externalising difficulties, these correlations were weak.

Of the candidate mediators: internalising and externalising difficulties; self-concept; parent-child conflict and feelings of safety in their home neighbourhood all met the criteria for mediation, that is, they were associated with the exposure and the outcome. Of those, only the indirect effect via self-concept and internalising difficulties mediated a significant percentage of the relationship between childhood adversity and recurring PEs. In the multivariate model, the two mediators together provided a partial mediation, explaining 12.97% of the relationship (OR 1.10, 95% CI 1.03–1.17) while individually internalising difficulties explained 7.01% (OR 1.05, 95% CI 1.03–1.07) and self-concept explained 5.96% (OR 1.04, 95% CI 1.02–1.07) of the total relationship. The direct effect remained significant (OR 1.86, 95% CI 1.15–3.00).

## Discussion

Using data from a large, nationally representative cohort, we have found a strong relationship between childhood adversity and recurring PEs, with at least a fifth of the incidence of recurring PEs attributable to childhood adversity. We therefore postulate that the known relationship between childhood adversity and PEs may be primarily driven by those who reported recurring PEs. Our second key finding is that self-concept and internalising difficulties significantly mediate the relationship between childhood adversity and recurring PEs.

The roles of affective dysregulation and cognitive processes in mediating the relationship between childhood adversity and recurring PEs are emphasised by our findings. Our results showed that internalising difficulties and self-concept are the only candidate mediators that explain a significant proportion of this relationship. Internalising difficulties and self-concept are conceptually similar and depressive symptoms and self-esteem have been found to be strongly correlated, even when items of overlap in measurement are excluded (Masselink et al., 2018). The roles of self-concept and internalising difficulties in this relationship are likely related but likely still represent distinct pathways between childhood adversity and recurring PEs.

Self-concept has been previously established as associated with PEs, with positive changes in self-concept reducing the risk of PEs and lower self-concept being associated with higher rates of PE (Healy et al., 2019b). The role of self-concept in mediating between childhood adversity and recurring PEs may be related to the role it plays in mediating between poly-victimisation and distress (Turner, Shattuck, Finkelhor, & Hamby, 2017). Turner et al., note that family and friend social support does not directly protect against distress and suggest that the influence of these factors may work primarily through self-concept (Turner et al., 2017). This would fit with findings that social support is protective against adolescent PEs in those exposed to poly-victimisation (Crush et al., 2018). In its mediating role, self-concept represents a key target for intervention to reduce the risk of recurrence of PEs in those who have experienced childhood adversity. Interventions in this age group to improve self-concept have been developed, however further work in those with specific risk-factors such as childhood adversity is still required, and it is not known whether changes in self-concept will have a downstream effect on PEs (Katz, Mercer, & Skinner, 2020).

This study builds on our previous work on mediators between childhood adversity and psychopathology in adolescence by exploring the specific relationships for PEs and linking them to internalising and externalising difficulties (Healy et al., 2021). Bidirectional relationships between internalising and externalising difficulties and PEs in adolescence have previously been found in this dataset (Healy, Coughlan, Clarke, Kelleher, & Cannon, 2020). That internalising difficulties explain a significant proportion of the relationship between childhood adversity and recurring PEs is in keeping with previous research findings for a role of depression in this relationship (Hafeez & Yung, 2020). The role that internalising difficulties play in the pathway from childhood adversity to recurring PEs may in part explain some of the relationships between recurring PEs and non-psychotic psychopathology.

In the supplementary analysis, neighbourhood safety was also found to be a significant mediator by both alternative methods (using a cumulative adversity measure or an item response theory approach). This may demonstrate a significant role for subsequent stressors ongoing at age 9 (as represented by a lack of safety in the child's current environment) or of negative appraisal of life circumstances at that time. It is also possible that children who do not find their neighbourhood to be safe at age 9 may be expressing paranoid thoughts or persecutory ideas, which are themselves a subcomponent of the PEs we are assessing.

Although internalising and externalising difficulties; self-concept; parent-child conflict and feelings of safety in their neighbourhood all met the criteria for mediation, only self-concept and internalising difficulties mediated a significant percentage of the relationship between childhood adversity and recurring PEs in our primary analysis. Together they account for only 13% of that relationship, though as much as 26.5% in the supplementary analysis. This is in part related to the inclusion of the neighbourhood safety variable, which remains significant in those analyses, as well as more of the overall influence of childhood adversity being captured by the less restrictive measures used in the supplementary analysis, particularly with the cumulative childhood adversity measure for which internalising difficulties explain nearly 12% of the relationship. While we have previously reported that parent-child conflict was the most important mediator in the relationship between childhood adversity and internalising and externalising difficulties, it does not explain a significant proportion of the relationship between childhood adversity and recurring PEs in this study, despite the significance of internalising difficulties in this relationship (Dhondt et al., 2019; Healy et al., 2021). In previous studies of the relationship between internalising difficulties and PEs, self-concept was a more prominent mediator than parent-child conflict for the relationship in early adolescence (Healy et al., 2020). The greater role of self-concept may be in part due to the timing in adolescence and the direction of the relationship being measured.

If recurring PEs are the driving force behind the relationship between childhood adversity and PEs, this may be suggestive of alternative processes or further components of mediation at play. In particular, recurring PEs may reflect persisting environmental risk factors or exposure to multiple environmental risks (as adversities cluster together, those with prior childhood adversity may be at increased ongoing risk) (Wigman et al., 2011). A recent study on predicting the persistence of hallucinations from childhood to adolescence failed to find significant differences in the prevalence of similarly parent-retrospectively reported childhood adversity at age 10 between recurring and transient PEs overall, though the highest prevalence of adversity was in the recurring PEs group (Steenkamp et al., 2021). This and recent evidence from another cohort study suggest that the risk factors for overall and for recurring PEs are the same, and severity or recurrence of exposure to adversity may be more relevant to the severity or recurrence of PEs (Rammos et al., 2021).

### Clinical implications

Self-concept has previously been identified as a target to reduce the risk of PEs (Healy et al., 2019b). These findings suggest there could be a benefit in child and adolescent services pre-emptively providing interventions to improve and maintain self-concept to those with at risk backgrounds. That internalising difficulties represent a similar target emphasises the importance of providing support for children already engaging with psychiatric services. Clinicians should keep risks associated with poor clarity of self-concept and internalising difficulties in mind when managing children and adolescents who have experienced childhood adversity, particularly as difficulties with self-concept and internalising may cause less conflict and less disruption at home and in the classroom, and not feature as prominently in presentations. The mediating role of these presentations demonstrates the importance of managing them at the time of presentation to prevent conversion to PEs during adolescence, increasing the risk of long-term psychiatric morbidity.

### Strengths and limitations

Using a large-scale nationally representative cohort has given us a large sample with longitudinal data collected over three time points, that includes validated measures and allows us to adjust for a number of possible confounds. Retention of 75% over three time points with reweighting for attrition assists in the reduction of sampling bias.

The limitations of this paper include a number of the measures relied on for our analysis, e.g. childhood adversity does not include information on experiences of abuse or neglect, nor ongoing adversity or poly-victimisation that may be present in this sample. Similarly we were limited in our confounds to assess the data as existed, so the childrens' ethnicity could not be included as a confound. Nor does our measure of PEs provide us with data on the frequency or severity of PEs. We are reliant on primarily parent-reported measures at age 9, and parent-reported measures of externalising and internalising difficulties may differ from child-reported ones, underestimating the prevalence of these mediators (van Roy, Groholt, Heyerdahl, & Clench-Aas, 2010). In some cases, there was no validated measure available in the age 9 data (such as for peer relationships, neighbourhood safety, diet and exercise participation) and we have relied on proxy measures which may not adequately capture social participation, weakening any conclusions that can be drawn from our findings on these mediators. Additionally, we were unable to undertake bootstrapping due to the weighting of the data, limiting our understanding of the accuracy of our inference.

Another limitation from the data is that questionnaire items were answered with variable completeness by participants. In our previous research using this dataset, we included a supplementary analysis where missing data was imputed and overall the change in the estimates were in the range of 0.01–0.05 (Dhondt et al., 2019). Given this, the effect of missing data on our overall results is hopefully minimal.

## Conclusion

There is a strong relationship between childhood adversity and recurring PEs in adolescence that is partially mediated by internalising difficulties and self-concept in childhood. Studies of interventions to improve self-concept and reduce the risk of internalising difficulties in young people who have experienced childhood adversity or trauma would be the next step. Further research on the risk factors for and mediators of this relationship between childhood adversity and recurring PEs may identify further targets for intervention to reduce the incidence of psychiatric illness and improve functioning in young adulthood.

## Supplementary material

For supplementary material accompanying this paper visit https://doi.org/10.1017/S003329172200071X. click here to view supplementary material
