# Supplementary material for: Childhood adversity and recurrence of psychotic experiences during adolescence: the role of mediation in an analysis of a population-based longitudinal cohort study
Source: Psychol Med. 2022 Mar 21;53(9):4046–54. doi: 10.1017/S003329172200071X (PMC10317802; doi:10.1017/S003329172200071X)
Supplement: Supplementary file 1 [file S003329172200071Xsup001.docx]

**Supplementary Materials**

*Supplementary childhood adversity analysis*

As in our previous work we ran supplementary analysis using alternative approaches to measuring childhood adversity; a cumulative measure and an item derived using item response theory. The cumulative measure variable treats all stressors as equally contributing degrees of adversity, which may not be accurate, and so as in our previous work we have conducted another analysis using 2-parameter item response theory (IRT), for a latent adversity score accommodating the cumulative effect of each stressor and the variability in severity.

*Supplementary analysis one: cumulative adversity measure*

The cumulative adversity variable (supplementary table 1) is broadly (with a dip at four or more) associated with increased risk of recurring PEs. Supplementary table 2 shows the relationship between individual items of our adversity measure and recurring PEs, only three of which are individually associated with PEs. It is worth noting here that the prominence of the relationship with ‘mental disorder in the immediate family’ demonstrates what may be an additional confounding effect from heritability. When you exclude these items there is no significant relationship with recurring PEs.

As shown in table 3, the cumulative measure is associated with all the mediators except exercise participation and diet factors. In the mediation analysis, internalising and self-concept remain significant mediators with the addition of the ‘feeling of safety in their home neighbourhood’ variable. In this model in which 26.5% of the relationship between childhood adversity and recurring PEs is explained, the direct effect becomes non-significant (OR: 1.16, 95% CI: 1.00-1.36).

Supplementary table 1: cumulative effects of stressors on recurring PEs as compared with no PEs

| No. stressors | % | Recurring PEs OR (95% CI) |
| --- | --- | --- |
| None | 21.3 | - |
| One or more | 78.7 | 1.80 (0.97-3.34) |
| Two or more | 44.7 | **1.72 (1.09-2.72)** |
| Three or more | 20.0 | **2.07 (1.24-3.47)** |
| Four or more | 8.1 | Non-significant |
| Five or more | 3.3 | **Significantly more** |
| Cumulative effect |  | **1.28 (1.15-1.41)** |
| Cumulative effect of stressors that do not have an individual effect on outcomes |  | 1.17 (0.66-2.09) |
| Emboldened statistics are significant at the p<0.05 level. Stressors are shown in the following table. Due to the small number of participants who report both individual CA items and recurrent PEs, several ORs describing groups of fewer than 30 participants that represent a statistical disclosure risk per GUI guidelines could not be included. | | |

Supplementary table 2: individual childhood adversity item prevalence and risk of recurring PEs as compared with no PEs

| Type of adversity | % reporting | OR (95% CI) |
| --- | --- | --- |
| Death of a parent | 2.4 | Non-significant |
| Death of a close family member | 43.2 | 0.93 (0.59-1.46) |
| Death of a close friend | 5.6 | Non-significant |
| Divorce or separation of parents | 14.1 | 1.78 (0.94-3.38) |
| Moving house | 41.4 | 1.11 (0.73-1.68) |
| Moving country | 9.6 | Non-significant |
| A stay in foster care | 1.2 | Non-significant |
| Serious illness or injury to the child | 5.1 | Non-significant |
| Serious illness or injury to a close family member | 13.7 | 1.51 (0.82-2.79) |
| Drug-taking or alcoholism in the immediate family | 3.2 | Non-significant |
| Mental disorder in the immediate family | 3.4 | **Significantly more** |
| Conflict between parents | 11.8 | **2.36 (1.35-4.14)** |
| A parent in prison | 0.9 | **Significantly more** |
| Other unspecified event | 1.7 | Non-significant |
| Emboldened statistics are significant at the p<0.05 level. Stressors are shown in the following table. Due to the small number of participants who report both individual CA items and recurrent PEs, several ORs describing groups of fewer than 30 participants that represent a statistical disclosure risk per GUI guidelines could not be included. | | |

Supplementary table 3: the relationship between cumulative CA, age 9 mediators and recurring PEs as compared with no PEs

|  | Prediction by cumulative adversity OR (95% CI) | Prediction of reoccurring PEs OR (95% CI) | Univariate indirect effect OR (95% CI) | % accounted for | Multivariate indirect effect OR (95% CI) | % accounted for |
| --- | --- | --- | --- | --- | --- | --- |
| **Candidate mediators associated with both exposure and outcome** | | | | | | |
| Internalising | **1.25 (1.14-1.36)** | **3.25 (1.87-5.64)** | **1.01 (1.01-1.04)** | **9.53** | **1.02 (1.01-1.03)** | **11.87** |
| Neighbourhood safety | **0.82 (0.71-0.94)** | **0.21 (0.11-0.41)** | **1.02 (1.00-1.03)** | **7.32** | **1.02 (1.01-1.03)** | **9.12** |
| Self-concept | **0.07 (0.04-0.10)*** | **1.34 (1.11-1.62)** | **1.02 (1.00-1.03)** | **8.13** | **1.04 (1.02-1.07)** | **5.51** |
| Externalising | **1.28 (1.17-2.09)** | **2.61 (1.30-5.28)** | 1.02 (1.00-1.04) | 8.76 | - | - |
| Parent-child conflict | **0.11 (0.08-0.14)*** | **1.24 (1.03-1.50)** | 1.02 (1.00-1.03) | 6.86 | - | - |
| **Candidate mediators not associated with both exposure and outcome** | | | | | | |
| Having more than one friend | **0.84 (0.75-0.93)** | 0.53 (0.27-1.01) | - | - | - | - |
| Parent-child positivity | **0.03 (0.00-0.06)*** | 1.02 (0.82-1.28) | - | - | - | - |
| Exercise frequency | 1.02 (0.96-1.08) | 1.15 (0.74-1.79) | - | - | - | - |
| Fruit and vegetable consumption | -0.02 (-0.01-0.05)* | 0.83 (0.68-1.02) | - | - | - | - |
| Refined carbohydrate consumption | 0.02 (-0.01-0.05)* | 1.21 (0.99-1.49) | - | - | - | - |
| *Asterisks indicate linear regression coefficients as opposed to ORs. Emboldened statistics are significant at the p<0.01 level. | | | | | | |

*Supplementary analysis two: Item response theory approach*

As in our previous work, a 2pl-IRT model was found to be preferable to a 1pl-IRT model. This 2pl-IRT model is based on two parameters: discrimination and difficulty, based on an item characteristic curve (ICC) showing the probability of a person endorsing a given item on the scale, given their level of the latent trait intended to be measured. Items with greater ‘difficulty’ are less frequently endorsed and are shifted to the right on the ICC – that is, they indicate a higher level of the latent trait. Discrimination is indicated by the slope of the ICC. Items with the same ‘discrimination’ are those with the same probability of being endorsed given the same level of the latent trait. Higher discrimination items show a greater difference between different levels of the latent trait. In supplementary table 4 the fit criteria and likelihood ratio test for these models is shown. In supplementary table 5 the parameters are shown with the prevalence of each item.

We generated a latent adversity score for each participant based on these discrimination and difficulty parameters (Mean: -0.09; SD: 0.63; Range: -0.64-3.79). The relationship between this latent variable, mediators and recurring PEs are shown in supplementary table 6. The overall relationship is significant (OR: 1.83; 95% CI: 1.41-2.37) and the direct effect continues to be significant when adjusting for mediators that explain 19.76% of the relationship in the multivariate model (OR: 1.51; 95% CI: 1.15-2.01). As in the cumulative model, ‘feeling of safety in the child’s home neighbourhood’ continues to be a significant mediator in this model.

Supplementary table 4: fit criteria and likelihood ratio tests for the 1- and 2pl models

| Model | Log likelihood | df | Chi diff (p-value) | AIC | BIC |
| --- | --- | --- | --- | --- | --- |
| 1-pl | -22922.52 | 15 | 1450.12 | 45875.04 | 45975.62 |
| 2-pl | -22261.92 | 28 | (>0.001) | 44579.83 | 44767.60 |

Supplementary table 5: the prevalence, discrimination and difficulty of each CA item

| Type of adversity | % reporting | Discrimination (95% CI) | Difficulty (95% CI) |
| --- | --- | --- | --- |
| Death of a parent | 2.4 | 0.14 (-0.36-0.63) | 26.79 (-68.41-122.00) |
| Death of a close family member | 43.2 | **-0.15 (-0.27- -0.03)** | **-1.83 (-3.25- -0.41)** |
| Death of a close friend | 5.6 | 0.18 (-0.07 – 0.42) | 15.98 (-6.21 – 38.18) |
| Divorce or separation of parents | 14.1 | **2.73 (2.13-3.33)** | **1.25 (1.14-1.36)** |
| Moving house | 41.4 | **0.81 (0.65-0.98)** | **0.48 (0.35-0.61)** |
| Moving country | 9.6 | **0.66 (0.47-0.85)** | **3.66 (2.69-4.62)** |
| A stay in foster care | 1.2 | **1.26 (0.96-1.57)** | **4.10 (3.35-4.86)** |
| Serious illness or injury to the child | 5.1 | **0.44 (0.23-0.66)** | **6.82 (3.59-10.05)** |
| Serious illness or injury to a close family member | 13.7 | **0.44 (0.28-0.66)** | **4.33 (2.78-5.89)** |
| Drug-taking or alcoholism in the immediate family | 3.2 | **1.70 (1.25-2.15)** | **2.69 (2.26-3.45)** |
| Mental disorder in the immediate family | 3.4 | **1.41 (1.08-1.74)** | **2.94 (2.43-3.45)** |
| Conflict between parents | 11.8 | **4.44 (2.85-6.02)** | **1.24 (1.12-1.36)** |
| A parent in prison | 0.9 | **1.83 (1.02-2.62)** | **3.40 (2.60-4.21)** |
| Other unspecified event | 1.7 | **0.67 (0.29-1.05)** | **6.39 (3.07-9.71)** |
| Emboldened statistics are significant at the p<0.05 level. Due to the small number of participants who report both individual CA items and recurrent PEs, several ORs describing groups of fewer than 30 participants that represent a statistical disclosure risk per GUI guidelines could not be included. | | | |

Supplementary table 6: the relationship between latent CA, age 9 mediators and recurring PE as compared with no PEs

|  | Prediction by latent adversity OR (95% CI) | Prediction of reoccurring PEs OR (95% CI) | Univariate indirect effect OR (95% CI) | % accounted for | Multivariate indirect effect OR (95% CI) | % accounted for |
| --- | --- | --- | --- | --- | --- | --- |
| **Candidate mediators associated with both exposure and outcome** | | | | | | |
| Internalising | **1.51 (1.27-1.79)** | **3.25 (1.87-5.64)** | **1.04 (1.01-1.08)** | **7.33** | **1.05 (1.03-1.07)** | **9.12** |
| Neighbourhood safety | **0.67 (0.52-0.86)** | **0.21 (0.11-0.41)** | **1.02 (1.00-1.03)** | **7.32** | **1.03 (1.02-1.04)** | **6.06** |
| Self-concept | **0.16 (0.1-0.22)*** | **1.34 (1.11-1.62)** | **1.04 (1.00-1.08)** | **6.8** | **1.02 (1.01-1.04)** | **4.58** |
| Externalising | **1.60 (1.16-2.06)** | **2.61 (1.30-5.28)** | 1.04 (1.00-1.08) | 6.48 | - | - |
| Parent-child conflict | **0.25 (0.19-0.30)*** | **1.24 (1.03-1.50)** | 1.03 (0.99-1.08) | 5.66 | - | - |
| **Candidate mediators not associated with both exposure and outcome** | | | | | | |
| Having more than one friend | **0.73 (0.60-0.89)** | 0.53 (0.27-1.01) | - | - | - | - |
| Parent-child positivity | 0.05 (-0.01-0.10)* | 1.02 (0.82-1.28) | - | - | - | - |
| Exercise frequency | 1.05 (0.93-1.18) | 1.15 (0.74-1.79) | - | - | - | - |
| Fruit and vegetable consumption | -0.06 (-0.11-0.00)* | 0.83 (0.68-1.02) | - | - | - | - |
| Refined carbohydrate consumption | 0.08 (-0.02-0.14)* | 1.21 (0.99-1.49) | - | - | - | - |
| *Asterisks indicate linear regression coefficients as opposed to ORs. Emboldened statistics are significant at the p<0.01 level. | | | | | | |

Supplementary table 7. Correlations between mediators

Table 1. Correlations coefficients between the mediators.

| Variable | Self-concept | Parent-child Conflict | Parent-child Positive | Fruit and Veg | Carbs | Neighborhood | Internalizing | Externalizing | Exercise |
| --- | --- | --- | --- | --- | --- | --- | --- | --- | --- |
| Self-concept | 1 |  |  |  |  |  |  |  |  |
| Parent-child Conflict | 0.17 | 1 |  |  |  |  |  |  |  |
|  | **<.001** |  |  |  |  |  |  |  |  |
| PIANTA Positive | 0.071 | 0.25 | 1 |  |  |  |  |  |  |
|  | **<.001** | **<.001** |  |  |  |  |  |  |  |
| Fruit and Veg | -0.06 | -0.07 | -0.05 | 1 |  |  |  |  |  |
|  | **<.001** | **<.001** | **<.001** |  |  |  |  |  |  |
| Carbs | 0.04 | 0.11 | 0.05 | -0.11 | 1 |  |  |  |  |
|  | **<.001** | **<.001** | **<.001** | **<.001** |  |  |  |  |  |
| Neighborhood | -0.16 | -0.05 | -0.05 | 0.04 | 0.01 | 1 |  |  |  |
|  | **<.001** | **<.001** | **<.001** | **<.001** | 0.55 |  |  |  |  |
| Internalizing | 0.15 | 0.27 | 0.13 | -0.08 | 0.09 | -0.00 | 1 |  |  |
|  | **<.001** | **<.001** | **<.001** | **<.001** | **<.001** | 0.73 |  |  |  |
| Externalizing | 0.15 | 0.37 | 0.15 | -0.06 | 0.10 | -0.11 | 0.21 | 1 |  |
|  | **<.001** | **<.001** | **<.001** | **<.001** | **<.001** | **<.001** | **<.001** |  |  |
| Exercise | -0.06 | -0.05 | -0.06 | 0.04 | -0.03 | 0.01 | -0.05 | -0.01 | 1 |
|  | **<.001** | **<.001** | **<.001** | **<.001** | **0.04** | 0.29 | **<.001** | 0.69 |  |
| Friends | -0.08 | -0.08 | -0.05 | 0.06 | 0.00 | 0.03 | -0.23 | -0.09 | 0.05 |
|  | **<.001** | **<.001** | **<.001** | **<.001** | 0.84 | **0.02** | **<.001** | **<.001** | **<0.001** |

*Emboldened value denote p<.05
